# Inferring plant microRNA functional similarity using a weighted protein-protein interaction network

**DOI:** 10.1186/s12859-015-0789-4

**Published:** 2015-11-04

**Authors:** Jun Meng, Dong Liu, Yushi Luan

**Affiliations:** 1 School of Computer Science and Technology, Dalian University of Technology, Dalian, Liaoning China; 2 School of Life Science and Biotechnology, Dalian University of Technology, Dalian, Liaoning China

**Keywords:** miRNA, Functional similarity, PPI, Gene Ontology

## Abstract

**Background:**

MiRNAs play a critical role in the response of plants to abiotic and biotic stress. However, the functions of most plant miRNAs remain unknown. Inferring these functions from miRNA functional similarity would thus be useful. This study proposes a new method, called PPImiRFS, for inferring miRNA functional similarity.

**Results:**

The functional similarity of miRNAs was inferred from the functional similarity of their target gene sets. A protein-protein interaction network with semantic similarity weights of edges generated using Gene Ontology terms was constructed to infer the functional similarity between two target genes that belong to two different miRNAs, and the score for functional similarity was calculated using the weighted shortest path for the two target genes through the whole network. The experimental results showed that the proposed method was more effective and reliable than previous methods (miRFunSim and GOSemSim) applied to *Arabidopsis thaliana*. Additionally, miRNAs responding to the same type of stress had higher functional similarity than miRNAs responding to different types of stress.

**Conclusions:**

For the first time, a protein-protein interaction network with semantic similarity weights generated using Gene Ontology terms was employed to calculate the functional similarity of plant miRNAs. A novel method based on calculating the weighted shortest path between two target genes was introduced.

**Electronic supplementary material:**

The online version of this article (doi:10.1186/s12859-015-0789-4) contains supplementary material, which is available to authorized users.

## Background

MicroRNAs (miRNAs) are single-stranded noncoding RNAs and are typically ~22 nucleotides long. These molecules are involved in post-transcriptional regulation and trigger targeted degradation of messenger RNA or inhibit translation [1, 2]. In plants, the expression of miRNA genes is a multistep process. First, the miRNA gene is initially transcribed as a primary miRNA sequence (pri-miRNA) by RNA polymerase II. Then, the pri-miRNA is processed into a hairpin sequence (precursor miRNA) by the endoribonuclease Dicer. Next, the loop region of the precursor miRNA is removed from the hairpin to form a miRNA duplex (miRNA:miRNA^*^). Finally, the miRNA^*^ strand is degraded, and the other miRNA strand, named the mature miRNA, is incorporated into the RNA-induced silencing complex (RISC) [3].

miRNAs that predominantly act as regulators of gene expression are involved in many plant biological processes such as development, nutrient homeostasis, biotic stress responses, abiotic stress responses and pathogen responses [3]. Previous studies have verified that groups of miRNAs are involved in many biological processes [4, 5]. Therefore, miRNAs involved in the same biological process should have identical or similar group functions. Currently, the number of miRNAs with functional annotations is limited, and the functions of some miRNAs are only partly known. Therefore, research on miRNA function has received increasing attention. In recent years, biologists have compared the functions of miRNA genes and predicted the potential functions of miRNAs based on the relationship between miRNAs with known molecular functions or associated with a specific stressor and those with unknown functions.

To date, only a few computational models have been available for inferring the functional similarity among miRNAs. In one report, functional similarity scores of human miRNAs were computed based on human miRNA-disease association data [6]. This computational method was implemented by measuring the similarity of miRNA-associated diseases structured as directed acyclic graphs and is similar to inferring the similarity of protein-coding genes by measuring the semantic similarity weights of Gene Ontology (GO) terms [7]. In the Online Mendelian Inheritance in Man (OMIM) disease similarity network, a random walk was applied to predict potential disease-miRNA associations under the assumption that functionally related miRNAs are often associated with phenotypically similar diseases [8]. The above two methods make full use of the associations among phenotypically similar diseases and obtained very satisfactory performance on the human data, but there are no disease similarity network data for plants, preventing the application of these strategies to plants. Because miRNAs are involved in biological processes through the regulation of their target transcripts, the functional similarity of miRNAs can be inferred by studying the associations of their target genes. In previous studies, several computational methods were proposed based on the associations between target genes. The simplest method used the proportion of the common target genes regulated by two miRNAs calculated by the Jaccard similarity measure [9]. Each plant miRNA regulates a small number of target genes, and the target gene sets of most plant miRNAs have no intersections; therefore, most of the calculation results from the Jaccard similarity measure are zeros. Therefore, this method is also not suitable for plants. A systematic method for studying the functional similarity of human miRNAs was proposed [10]. The functional similarity between two miRNAs was quantified by measuring the semantic similarity weights of the GO terms between two miRNA target genes. A new definition called a co-regulating functional module was introduced [11]. The GO categories of the target gene sets of each pairwise set of miRNAs were used to test the significance of their co-regulated target genes using a hypergeometric test. The co-regulating functional modules were established using a protein-protein interaction network (PPIN). miRNAs that shared at least one co-regulating functional module were considered to have similar functions. The shortcomings of this method are that the results are only 0 or 1 and that it cannot generate numerical results to measure the level of similarity. Another method used a target gene network to measure the functional similarity of miRNAs. This method considered both the target site accessibility and the interactive context of the target genes in a functional gene network constructed with semantic similarity weights generated using the GO terms of the target genes [12]. Because the GO annotations are incomplete, the functional gene network constructed may not be as realistic as those of networks confirmed by experimental data, such as PPI networks. PPINs have been widely used to predict protein function [13], protein complexes [14], and gene functional similarity [15]. Furthermore, the functional similarity scores of human miRNAs were computed using a PPIN that quantified the associations between the miRNAs based on their targeting propensities and protein connectivity in an integrated PPIN [16].

Most of the existing computational methods have been designed specifically for human. Based on the above analysis, few of them can be used for plants. It is thus necessary to develop an effective and stable computational method for calculating functional similarity scores of plant miRNAs. This study proposes a novel computational method, called PPImiRFS, to obtain the functional similarity scores of miRNA pairs based on a PPIN with semantic similarity weights generated using GO terms and graph theoretic properties. The proposed method is available for download at our supporting website: https://github.com/kobe-liudong/PPImiRFS. The miRNA families, miRNA clusters and experimentally verified miRNAs associated with biotic and abiotic stress responses in *Arabidopsis thaliana* (*A. thaliana*) were used to evaluate and validate the performance of our method. Furthermore, a comparative analysis showed that our method was more effective and reliable than two widely used computational methods (miRFunSim [16] and GOSemSim [10]).

## Methods

### *A. thaliana* miRNA and mRNA

All of the *A. thaliana* mature miRNA sequences, *A. thaliana* miRNA families and genome coordinates of the miRNAs were downloaded from miRBase [17] (Release 21, June 2014). This release contains 427 mature sequences, 47 families containing more than one miRNA and 30 clusters (with 10 kb as the maximum inter-miRNA distance for two miRNA genes to be clustered together) [18]. The *A. thaliana* candidate mRNAs were obtained from the TAIR database, which includes all of the transcribed sequences [19] (Release 10). The families and clusters of *A. thaliana* miRNAs are presented in Additional file 1 and Additional file 2, respectively.

### *A. thaliana* miRNAs in response to stress

There is no publicly available database of *A. thaliana* miRNAs related to their response to abiotic and biotic stress; thus, we obtained 126 experimentally verified *A. thaliana* miRNAs associated with the stress response, including 12 types of abiotic stress and 3 types of biotic stress, by referring to 25 reports listed in Additional file 3, which also presents the 126 experimentally verified *A. thaliana* miRNAs that respond to various types of stress.

### Method description

**Fig. 1:**
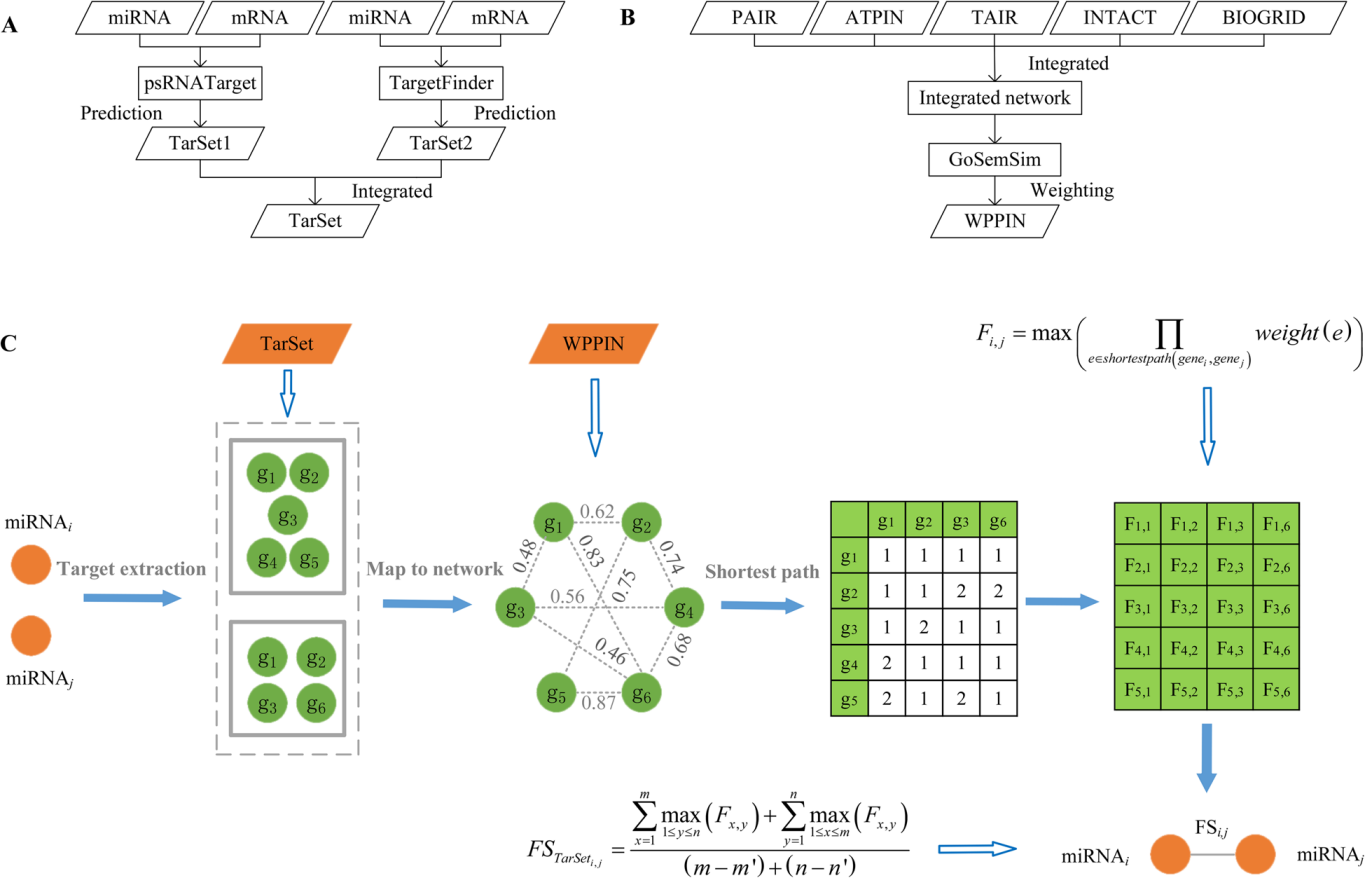
Flow chart of PPImiRFS. **a** Prediction of target genes. **b** Construction of a weighted integrated protein-protein interaction network. **c** Calculating the miRNA functional similarity scores. The filled arrows represent the workflow of the PPImiRFS method, and the closed arrows represent the computational methods or datasets used in each step

The flow chart for PPImiRFS is shown in Fig. 1. First, a weighted protein-protein interaction network (WPPIN) was constructed by combining a PPIN with GO term semantic similarity weights. Second, the target genes of the miRNAs were predicted with two tools, psRNATarget [20] and Targetfinder [21], using their default settings. Third, the functional similarity score between the target gene sets of each miRNA pair of interest was calculated based on the WPPIN and a modified weighted breadth-first search (BFS) algorithm. Then, we obtained a functional similarity matrix for the target gene sets of the miRNA pairs. Finally, the functional similarity scores of each pair of miRNAs were calculated using the functional similarity matrix of the target gene sets and a modified method that is based on best-match average (BMA) [22, 23].

### Integrated PPIN

The *A. thaliana* PPIN data were download from TAIR (http://www.arabidopsis.org/) [19], AtPIN (http://bioinfo.esalq.usp.br/web/) [24], PAIR (http://www.cls.zju.edu.cn/pair/) [25], BioGRID (http://thebiogrid.org/) [26] and IntAct (http://www.ebi.ac.uk/intact/) [27]. These databases have all been widely used in other studies on *A. thaliana*. We integrated the data from these various databases and derived the non-redundant *A. thaliana* PPIN containing 88,484 interactions between 10,985 proteins. The topological characteristics of the *A. thaliana* PPIN and the percentage of each dataset in the integrated PPIN are shown in Table 1.

**Table 1:** Topological characteristics of the *A. thaliana* PPIN

Database	Proteins No. (%)	Interactions No. (%)	Degree		
			Max	Min	Average
TAIR	7,115 (64.8 %)	70,699 (79.9 %)	737	1	19.87
AtPIN	2,807 (25.6 %)	6,204 (7.0 %)	134	1	4.42
PAIR	2,776 (25.3 %)	5,619 (6.4 %)	131	1	4.05
BioGRID	6,943 (63.2 %)	16,463 (18.6 %)	1297	1	4.74
IntAct	4,172 (38.0 %)	9,480 (10.7 %)	259	1	4.54
Integrated	10,985 (100 %)	88,484 (100 %)	1304	1	16.11

### Construction of the WPPIN

The weights of the PPIN were computed by measuring the functional similarity of the target genes based on the semantic similarity of their GO terms. The functional similarity weights were calculated by GOSemSim [28], an R package that has implemented the compute methods of the semantic similarity. GOSemSim supports 19 species, including *A. thaliana*, human, mouse, and yeast. For PPImiRFS, we used geneSim in the GOSemSim package to calculate the semantic similarity between two target genes. In geneSim, a graph-based semantic similarity measurement method [7] is used. The GO data used in the experiment were collected and processed by the GOSemSim and its version is 2.14.0. Because GO is composed of three orthogonal ontologies, molecular function (MF), biological process (BP), and cellular component (CC), we calculated the semantic similarity weights of the GO terms of a pair of target genes using each of the three orthogonal ontologies separately and then constructed three WPPINs.

### Prediction of the miRNA target genes

A few experimentally validated target genes of *A. thaliana* miRNAs are available. We obtained miRNA target genes using the prediction method described below. In contrast to animals, particularly humans, most miRNAs in plants have only a few target genes, likely because of the near-perfect complementarity between plant miRNAs and their target genes. To prevent the bias produced by individual prediction methods and obtain more satisfactory results, the target genes of all of the miRNAs were predicted using two widely used plant miRNA target gene prediction tools, psRNATarget and Targetfinder, using their default settings. The prediction results were integrated by union, and any redundancies were removed. In our previous study on miRNA target gene prediction, we also used multiple tools (including psRNATarget) to prevent bias and obtain more satisfactory results [29]. The present results are summarized in Table 2.

**Table 2:** Results of *A. thaliana* miRNA target prediction

Tools	No. of miRNAs	No. of Targets			
		Sum	Average	Max	Min
psRNATarget	415	3,461	8.33	25	1
Targetfinder	397	7,372	18.57	1,741	1

### Functional similarity of target gene sets

A novel network-based weighted shortest path method was proposed to calculate the functional similarity between two target gene sets.

Given the target gene sets of two miRNAs, miRNA_*i*_ and miRNA_*j*_, we extract a target gene from each miRNA and calculate the functional similarity score for the two target genes. All of the shortest paths between the two target genes are obtained from the WPPIN. We use a modified weighted BFS to search for the shortest paths. This modified weighted BFS is more efficient in the weighted networks than the classical BFS. In the modified weighted BFS, we represent these shortest paths as a tree and prune the tree based on the accumulated weights during its growth. The functional similarity score of the pair of target genes is calculated using a best average accumulated weight method as follows:

1 $$ {F}_{i,j}= \max \left({\displaystyle \prod_{e\in shortestpath\left(gen{e}_i,gen{e}_j\right)} weight(e)}\right), $$

where *n* is the number of edges in the shortest path. The function max(*x*) means that *F*_*i, j*_ is the maximum of all of the results calculated by the average accumulated weight method when there is more than one shortest path between *gene*_*i*_ and *gene*_*j*_ in the WPPIN. *F*_*i, j*_ is equal to 1 when *gene*_*i*_ and *gene*_*j*_ are equivalent.

The functional similarity scores of all target gene pairs from the two target gene sets are obtained to form a functional similarity matrix, and the functional similarity score for the comparison between the target gene sets is calculated based on that functional similarity matrix using a modified BMA method, which is defined as follows:

2 $$ F{S_{TarSet}}_{{}_{i,j}}=\frac{{\displaystyle \sum_{x=1}^{m-m\hbox{'}}\underset{1\le y\le n-n\hbox{'}}{ \max}\left({F}_{x,y}\right)}+{\displaystyle \sum_{y=1}^{n-n\hbox{'}}\underset{1\le x\le m-m\hbox{'}}{ \max}\left({F}_{x,y}\right)}}{\left(m-m\hbox{'}\right)+\left(n-n\hbox{'}\right)}, $$

where *m* and *n* are the number of target genes of miRNA_*i*_ and miRNA_*j*_, respectively, and *n*’ and *m*’ are the number of target genes that are not included in the WPPIN.

## Results and discussion

### Functional similarity of the miRNAs in the same family or cluster

**Fig. 2:**
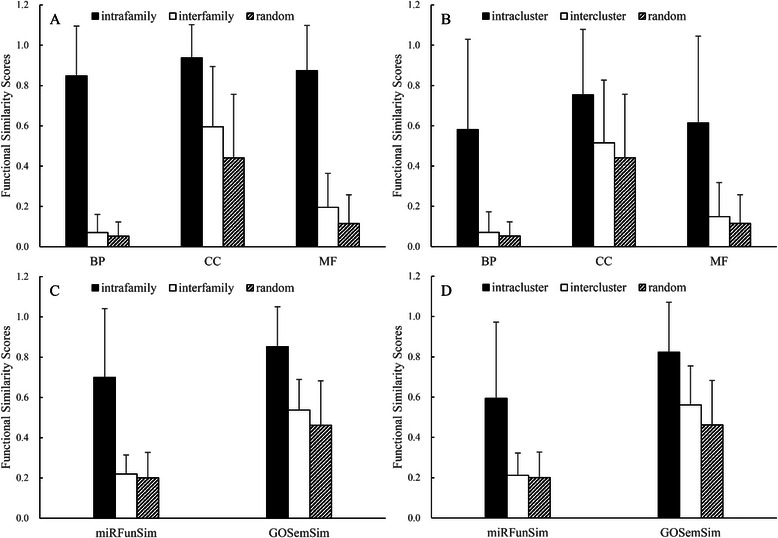
Performance evaluation of PPImiRFS on miRNA family and cluster data. **a** Results of family data based on BP, CC and MF terms calculated with PPImiRFS. **b** Results of cluster data based on BP, CC and MF terms calculated with PPImiRFS. **c** Results of family data calculated with miRFunSim and GOSemSim. **d** Results of cluster data calculated with miRFunSim and GOSemSim

Mature miRNAs in the same family exhibit sequence similarity and have completely identical seed regions for miRNA target recognition [30]. Therefore, the functions of the miRNAs in the same family are likely to be more similar than the functions of miRNAs in different families. Accumulating evidence supports this phenomenon [6, 11, 15]. To evaluate the reliability of the functional similarity scores computed by the PPImiRFS method, we divided all of the *A. thaliana* miRNAs into three classes: intrafamily, interfamily and randomly selected miRNA pairs (which are from among the 91378 miRNA pairs, excluding the miRNA pairs in the intrafamily, interfamily, intracluster and intercluster classes; there are 47 families that contain 158 miRNAs and 30 clusters that contain 74 miRNAs). The PPImiRFS method was then applied to compute the functional similarity scores of the miRNAs within each of the three classes. Because three WPPINs have been constructed based on three orthogonal ontologies (GO types), the above results should be calculated on the three WPPINs separately. The computed functional similarity scores based on BP, CC and MF terms are shown in Fig. 2a. We further studied the differences among the functional similarity scores of intrafamily, interfamily and randomly selected miRNA pairs. These functional similarity scores demonstrate significant differences (Kruskal-Wallis, df = 2; results are shown in Table 3). The functional similarity scores of the miRNAs in the intrafamily group are significantly higher than those in the interfamily and randomly selected miRNA groups (Wilcoxon rank-sum test; results are shown in Table 3). The similarity score matrix for all 91378 possible pairs of the *A. Thaliana* miRNA dataset is provided in Additional file 4.

**Table 3:** Statistical analysis results of functional similarity of the intrafamily, interfamily and randomly selected miRNAs

Methods	Intra-inter-random (p-value)	Intra-inter (p-value)	Intra-random (p-value)
PPImiRFS (BP)	0.0E0	1.41E-170	5.51E-182
PPImiRFS (CC)	0.0E0	1.42E-138	2.39E-162
PPImiRFS (MF)	0.0E0	9.28E-165	1.69E-178
miRFunSim	1.2354E-155	1.06E-139	2.82E-132
GOSemSim	0.0E0	1.40E-104	2.19E-117

Many miRNAs are located in close proximity to each other in genomes, forming clusters. Previous studies have suggested that miRNAs in the same cluster are often located in a polycistron and display homogeneous expression patterns [31], suggesting that the functions of these clustered miRNAs may be similar or identical. Therefore, miRNA cluster patterns were also studied with PPImiRFS using the same research method applied to miRNA families. The final results based on BP, CC and MF terms are shown in Fig. 2b. Statistical analysis reveals that the functional similarity scores among the miRNAs in the intracluster, intercluster and randomly selected miRNA pairs are also significantly different (Kruskal-Wallis, df = 2; results are shown in Table 4). The functional similarity scores of the miRNA pairs within the intracluster group are significantly higher than those of the intercluster and randomly selected miRNA groups (Wilcoxon rank-sum test; results are shown in Table 4).

**Table 4:** Statistical analysis results of functional similarity of the intracluster, intercluster and randomly selected miRNAs

Methods	Intra-inter-random (p-value)	Intra-inter (p-value)	Intra-random (p-value)
PPImiRFS (BP)	1.5538E-39	4.04E-15	3.88E-18
PPImiRFS (CC)	7.2078E-48	1.57E-11	3.20E-15
PPImiRFS (MF)	2.0064E-30	9.86E-14	8.14E-16
miRFunSim	9.0311E-22	9.54E-18	1.07E-16
GOSemSim	3.81E-140	1.28E-13	1.57E-20

To verify our result, the other two methods (miRFunSim [16] and GOSemSim [10]) were applied to the above experiment, and the results based on family and cluster data are shown in Fig. 2c and d. The functional similarity scores among the three classes of miRNA pairs are significantly different. The statistical analysis results of these methods based on family and cluster data are also shown in Tables 3 and 4, respectively.

In conclusion, the above two methods produce the same results as those obtained using PPImiRFS and clearly verify the utility of PPImiRFS.

### Functional similarity of miRNAs responding to identical types of stress

**Fig. 3:**
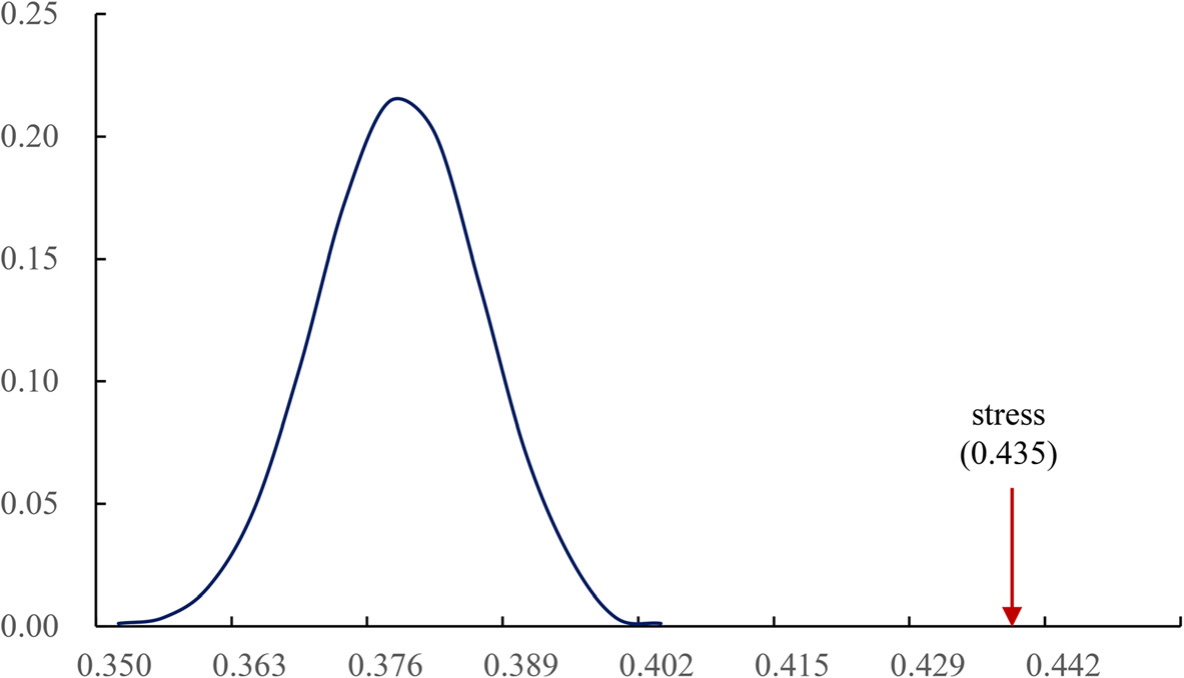
Average functional similarity scores of miRNA pairs in response to identical types of stress and randomly selected miRNA pairs. The red arrow represents the average functional similarity score of the miRNAs in response to identical types of stress. The normal distribution curve represents the distribution of the average functional similarity scores of the negative test datasets

In this study, we hypothesized that miRNAs responding to identical abiotic or biotic stresses are likely to have similar functions. To test our hypothesis, two classes of test datasets were generated: a positive test set with 324 miRNA pairs responding to identical abiotic or biotic stresses, from among 12 abiotic and 3 biotic stresses, and a negative test set with 324 miRNA pairs not responding to identical abiotic or biotic stresses. To obtain more objective results, we generated 50 negative test sets. The functional similarity scores of the miRNAs within these two classes of test sets were computed using the PPImiRFS method. The statistical analyses are shown in Fig. 3. The results support our hypothesis: the miRNAs responding to identical types of stress have greater functional similarity scores than those not responding to identical types of stress.

### Performance evaluation of PPImiRFS

**Fig. 4:**
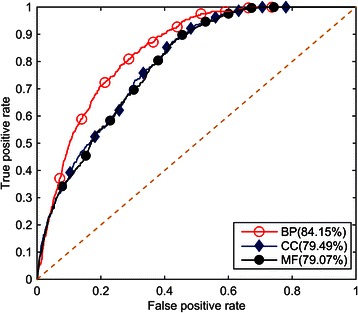
ROC curves from PPImiRFS based on the three orthogonal ontologies

To evaluate the performance of the proposed PPImiRFS method for scoring the functional similarity between miRNAs, the ability of our method to identify functional similarity was tested on experimentally verified miRNA-stress association data. First, 126 high quality, experimentally verified miRNA-stress associations were manually extracted from the literature. The miRNA pairs responding to identical types of stress were regarded as the positive test cases. A miRNA pair was composed of any two different *A. thaliana* miRNAs, and a total of 91378 miRNA pairs were obtained. The miRNA pairs in the same family, in the same cluster and in response to the same types of stress were excluded from the 91378 miRNA pairs. The remaining pairs were used as negative test cases. For each positive test case, 99 negative test cases were randomly selected from the above negative test cases, and the functional similarity scores of all cases were calculated with PPImiRFS. Next, we prioritized the computed scores of each positive test case with those of the negative test cases. Therefore, for each positive test case, a prioritization list of 100 miRNA pairs was generated. In total, 324 ranking lists were obtained, each with 100 prioritizations. Additionally, the true positive (TP) and false positive (FP) rates were both calculated at different thresholds based on the 324 ranking lists. The true positive rate (also called the sensitivity or recall rate in some fields) measured the proportion of the actual positives that were correctly identified as such, i.e., the proportion of the positive test cases that were ranked above a given threshold. The specificity (occasionally called the true negative (TN) rate) measured the proportion of negatives that were correctly identified as such, i.e., the proportion of negative test cases that were ranked lower than a given threshold. For example, if the threshold is 5, the TP is the proportion of real positives that are ranked above 5 in 324 lists, and the TN is the proportion of negatives that are ranked lower than 5. If there are 10 thresholds, there are 10 sets of TPs and TNs. Finally, a receiver operating characteristic (ROC) curve was plotted based on the results of the true positive and false positive rates, and the area under the curve (AUC) was calculated. The AUC was regarded to be a standard measure of the performance of PPImiRFS. If the value of the AUC was 100 %, the scores of the positive test cases were all ranked first in the ranking lists. A higher AUC value was indicative of higher PPImiRFS performance. AUC values were calculated based on each of the three constructed WPPINs. The proposed PPImiRFS method achieved AUC values of 84.15 %, 79.49 % and 79.07 % based on BP, CC and MF terms, respectively. The experimental results of our performance evaluation suggest that the PPImiRFS method can recover miRNA pairs responding to identical types of abiotic and biotic stress and efficiently quantify the relationship between the miRNAs. These results also reveal that the performance of PPImiRFS on the WPPIN weighted using the BP term exceeded that using MF or CC terms. The three ROC curves are shown in Fig. 4. In the next section, the results based on BP terms will be compared with existing methods.

### Comparison with existing similar methods

Recently, several computational methods have been proposed for quantifying the functional similarity scores of miRNAs. In this section, we selected two methods, miRFunSim and GOSemSim, for comparison with the proposed PPImiRFS method. miRFunSim is used to calculate the functional similarity between miRNAs based on the PPI data, and it only utilizes the structural features of PPI networks. One report has found that weighted PPI networks are more effective than unweighted PPI networks [32]. Because the GO data are incomplete, there are many null values in the result of GOSemSim, thereby affecting its performance. The proposed PPImiRFS not only considers the structural features of the PPI network but also includes the GO similarity weighting, which may allow it to overcome the deficiencies present in the above two methods.

**Fig. 5:**
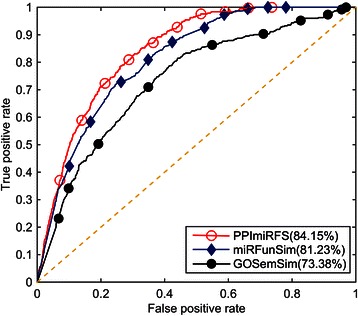
ROC curves from the three computational methods

By analyzing the ROC curves and corresponding AUC values, these three methods were compared. The miRFunSim and GOSemSim methods were tested on 126 high quality, experimentally verified *A. thaliana* miRNA-stress associations to calculate the functional similarity scores for every miRNA pair associated with identical types of stress. The comparison, shown in Fig. 5, demonstrates that PPImiRFS performs better than miRFunSim and GOSemSim.

**Fig. 6:**
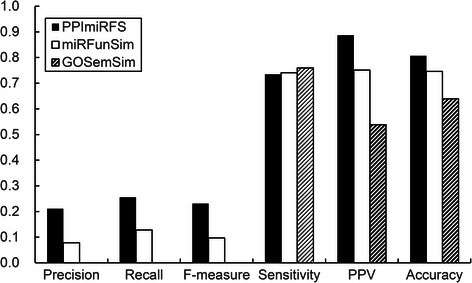
Comparative performance of ClusterONE based on the networks constructed with PPImiRFS, miRFunSim and GOSemSim

**Fig. 7:**
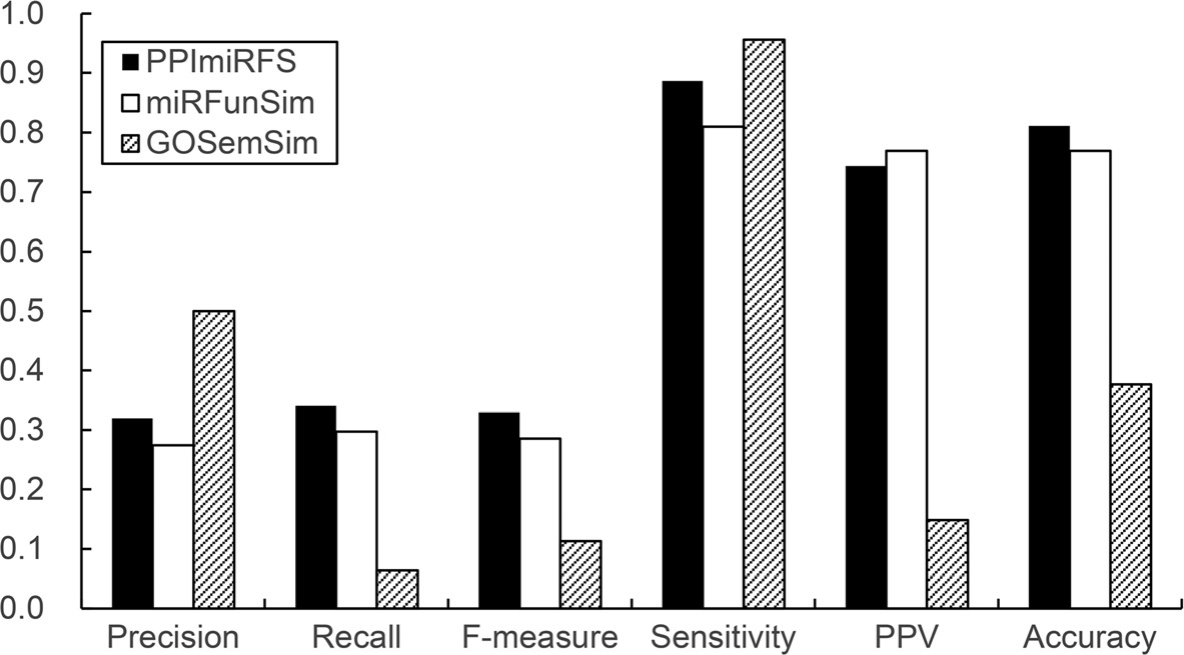
Comparative performance of the Connected Component Clusters based on the networks constructed with PPImiRFS, miRFunSim and GOSemSim

Similarity scores calculated using the three methods were used along with various clustering algorithms to cluster 427 miRNA sequences. The clustering results can be evaluated by using the 47 families as benchmark clusters. The performances of the three methods can be compared using the assessment results. The functional similarity scores of the 91378 miRNA pairs constructed from the 427 miRNAs were calculated by PPImiRFS, miRFunSim and GOSemSim, and then three weighted miRNA networks were constructed. Through an experiment comparing eight network clustering algorithms in clusterMaker [33] (Affinity Propagation Cluster, AutoSOME Network Clustering, Cluster Fuzzifier, Connected Components Cluster, Fuzzy C-Means Cluster, MCL Cluster, SCPS Cluster and Transitivity Clustering) and ClusterONE [34], we discovered that ClusterONE and Connected Components Cluster could obtain better results than the other clustering algorithms, and these two methods were therefore selected to cluster the 427 miRNAs. The basic parameters used for ClusterONE were as follows: for PPImiRFS and miRFunSim, the minimum size was 2, and the minimum density was 0.45; for GOSemSim, the minimum size was 1, and the minimum density was 0.85. ClusterONE predicted 57, 77 and 75 clusters for PPImiRFS, miRFunSim and GOSemSim, respectively. The edge weight cutoff values of Connected Components Cluster were 0.4, 0.55 and 0.9 for PPImiRFS, miRFunSim and GOSemSim, respectively. The numbers of clusters predicted with Connected Components Cluster for PPImiRFS, miRFunSim and GOSemSim were 50, 51 and 6, respectively. The evaluation metrics used by reference [35] were then applied to evaluate the cluster results. The evaluation metrics comprise precision, recall, F-measure, sensitivity, positive predictive value and accuracy. The evaluations of ClusterONE and Connected Components Cluster are shown in Figs. 6 and 7.

Figure 6 shows the comparisons of the PPImiRFS with other methods when ClusterONE is used. The proposed method outperformed the two previous methods, with the exception of its slightly lower sensitivity. Figure 7 shows the comparisons of the PPImiRFS with other methods when Connected Component Cluster is used. Although the highest precision and sensitivity were achieved in the network constructed with GOSemSim, this occurred because the number of clusters predicted was very small, including an impossibly large cluster containing 393 miRNAs. Therefore, most of the miRNAs in the benchmark clusters were included in this very large cluster, giving a very high sensitivity, and most of the members of other clusters were in the same family, meaning that they appeared in the benchmark clusters, giving a relatively high precision. Therefore, the network computed with GOSemSim was not truly better than those generated using PPImiRFS and miRFunSim.

In conclusion, the PPImiRFS method is more effective and reliable for quantifying the relationships between miRNAs than other available similar methods.

### Case study

In this section, we quantified the relationship between miRNAs in response to high salt content (abiotic stress) and the TMV-Cg virus (biotic stress) using the PPImiRFS method. The miRNAs responding to TMV-Cg were divided into two sets: the seed miRNAs and the test miRNAs. We combined the test mRNAs with the remaining *A. thaliana* miRNAs (with the exception of the miRNAs responding to TMV-Cg) as the final test miRNA set. Next, functional similarity scores were calculated between every miRNA in the seed miRNA set and every miRNA in the final test miRNA set. Finally, we ranked all of the miRNA pairs according to their scores. We retrieved the most miRNAs (with the exception of miR823) when we set the threshold to 0.5. We also predicted several new miRNAs that are likely to respond to the TMV-Cg virus, including miR165 [36, 37], miR156 [34, 38], miR418, miR160 [36, 38], and miR393 [36, 37, 39]. Next, the same experimental method was used on miRNAs responding to high-salt conditions. We retrieved all of these miRNAs with a threshold of 0.5. We also predicted several new miRNAs that are likely to respond to high-salt conditions, including miR418, miR166 [36, 40], miR160 [36, 38], miR841 [41], miR169 [37, 42, 43]. Although these new miRNAs have not been reported to respond to high-salt conditions or TMV-Cg in the miRBase database, several of them have been verified to respond to other types of stress. These cases will be further verified by biological experiments in the future. The partial results are shown in Table 5, and the complete results are available in Additional file 5.

**Table 5:** Top 5 prediction results for miRNAs responding to high-salt conditions and TMV-Cg stress

Stress	miRNA	Score
High-salt	ath-miR418	0.932
	ath-miR166	0.929
	ath-miR160	0.908
	ath-miR841	0.892
	ath-miR169	0.816
TMV-Cg	ath-miR165	1.000
	ath-miR156	0.939
	ath-miR418	0.932
	ath-miR160	0.908
	ath-miR8177	0.899

### Availability of PPImiRFS

To our knowledge, most of the existing methods mentioned previously have not been implemented as publicly available software packages. Therefore, their availability is limited. In this study, we not only introduced a novel computing method but also implemented a publicly available software package. This software package is composed of a main program, data pre-processing programs, and *A. thaliana* data. PPImiRFS is a console application programmed in C++, and the data pre-processing programs are implemented in Perl and R. The target gene sets of the miRNAs of the species to be inferred and the WPPIN data are required before the software can be run. The current version of the software package includes the necessary datasets for *A. thaliana*, and we will integrate datasets from additional species into future versions of the software. If users are interested in applying the current version of the software package to other species, all the necessary programs for generating the required datasets are provided. To use the software, users only need to input a file that includes their miRNA pairs of interest. The functional similarity scores of these miRNA pairs will be calculated automatically, and a file will be created that contains all of the functional similarity scores of the miRNA pairs in the input file. Our software was tested on a PC (2.5 GHz cup, 2 GB RAM) and required 0.13 h, 1.11 h and 6.00 h to finish with input files of 100, 1000 and 5000 miRNA pairs, respectively. The software is available at https://github.com/kobe-liudong/PPImiRFS.

## Conclusions

In this study, we proposed a novel computational method to quantify the functional similarity between a pair of plant miRNAs based on a PPIN with GO term semantic similarity weights. For the convenience of other researchers, we implemented our proposed method as a publicly available software package for local use. This study revealed that the functions of miRNAs responding to the same type of stress (abiotic or biotic) appeared more similar using the proposed method than those of miRNAs not responding to the same type of stress. By computing the functional similarity scores of intrafamily, interfamily and randomly selected miRNAs and intracluster, intercluster and randomly selected miRNAs, the miRNAs in the same family or cluster were shown to have higher functional similarity scores. These results suggest that our method can correctly identify the functional similarities and differences between miRNAs in different groups. Furthermore, in a comparison with other similar computational methods, our proposed method achieved the most effective and reliable performance.

Qualifying the functional similarity of miRNAs is based on a PPIN and predicted target gene sets, and the utilized plant PPIN has very low coverage and is often associated with high rates of false positives and false negatives. In addition, the predicted targets often have high false positive rates. Thus, our method will achieve higher performance as the quality of the PPIN increases, and improved target prediction methods are proposed. Lastly, PPImiRFS can be applied to any plant species with a PPIN and GO data.

## Additional files

Additional file 1: Table S1. The families of the *A. thaliana* miRNAs. (DOCX 17 kb)
Additional file 2: Table S2. The clusters of the *A. thaliana* miRNAs. (DOCX 18 kb)
Additional file 3: Table S3. The experimentally verified *A. thaliana* miRNAs that respond to different types of stress. (DOCX 29 kb)
Additional file 4:
**The similarity score matrix for all 91378 possible pairs of the** ***A. Thaliana*** **miRNA dataset.** (XLSX 1456 kb)
Additional file 5: Table S4. The complete results for the miRNAs responding to high-salt conditions and TMV-Cg stress. (DOCX 17 kb)
