# Supplementary material for: Inferring plant microRNA functional similarity using a weighted protein-protein interaction network
Source: BMC Bioinformatics. 2015 Nov 4;16:361. doi: 10.1186/s12859-015-0789-4 (PMC4634583; doi:10.1186/s12859-015-0789-4)
Supplement: Additional file 1: Table S1. — The families of the A. thaliana miRNAs. (DOCX 17 kb) [file 12859_2015_789_MOESM1_ESM.docx]

| Table S1. The families of *A.thaliana* miRNAs | | | |
| --- | --- | --- | --- |
| Family No. | miRNA | Family No. | miRNA |
| 1 | ath-miR165a-3p  ath-miR165b  ath-miR166a-3p  ath-miR166b-3p  ath-miR166c  ath-miR166d  ath-miR166e-3p  ath-miR166f  ath-miR166g | 2 | ath-miR156a-5p  ath-miR156b-5p  ath-miR156c-5p  ath-miR156d-5p  ath-miR156e  ath-miR156f-5p  ath-miR157a-5p  ath-miR157b-5p  ath-miR157c-5p  ath-miR157d  ath-miR156g  ath-miR156h |
| 3 | ath-miR159a  ath-miR159b-3p  ath-miR319a  ath-miR319b  ath-miR159c  ath-miR319c | 4 | ath-miR169h  ath-miR169i  ath-miR169j  ath-miR169k  ath-miR169l  ath-miR169m  ath-miR169n |
| 5 | ath-miR399a  ath-miR399b  ath-miR399c-3p  ath-miR399d  ath-miR399e  ath-miR399f | 6 | ath-miR395a  ath-miR395b  ath-miR395c  ath-miR395d  ath-miR395e  ath-miR395f |
| 7 | ath-miR167a-5p  ath-miR167b  ath-miR167d | 8 | ath-miR170-3p  ath-miR171a-3p  ath-miR171b-3p  ath-miR171c-3p |
| 9 | ath-miR160a-5p  ath-miR160b  ath-miR160c-5p | 10 | ath-miR172a  ath-miR172b-3p  ath-miR172c  ath-miR172d-3p  ath-miR172e-3p |
| 11 | ath-miR169a-5p  ath-miR169b-5p  ath-miR169c  ath-miR169d  ath-miR169e  ath-miR169f-5p  ath-miR169g-5p | 12 | ath-miR164a  ath-miR164b-5p  ath-miR164c-5p |
| 13 | ath-miR396a-5p  ath-miR396b-5p | 14 | ath-miR168a-5p  ath-miR168b-5p |
| 15 | ath-miR393a-5p  ath-miR393b-5p | 16 | ath-miR394a  ath-miR394b-5p |
| 17 | ath-miR390a-5p  ath-miR390b-5p | 18 | ath-miR398a-3p  ath-miR398b-3p  ath-miR398c-3p |
| 19 | ath-miR397a  ath-miR397b | 20 | ath-miR162a-3p  ath-miR162b-3p |
| 21 | ath-miR447a-3p  ath-miR447b  ath-miR447c-3p | 22 | ath-miR405a  ath-miR405b  ath-miR405d |
| 23 | ath-miR158a-3p  ath-miR158b | 24 | ath-miR854a  ath-miR854b  ath-miR854c  ath-miR854d  ath-miR854e |
| 25 | ath-miR845a  ath-miR845b | 26 | ath-miR2111a-5p  ath-miR2111b-5p |
| 27 | ath-miR2933a  ath-miR2933b | 28 | ath-miR774a  ath-miR774b-5p |
| 29 | ath-miR773a  ath-miR773b-3p | 30 | ath-miR841a-5p  ath-miR841b-5p |
| 31 | ath-miR781a  ath-miR781b | 32 | ath-miR858a  ath-miR858b |
| 33 | ath-miR3932a  ath-miR3932b-3p | 34 | ath-miR5020a  ath-miR5020b  ath-miR5020c |
| 35 | ath-miR782  ath-miR2934-5p | 36 | ath-miR5645a  ath-miR5645b  ath-miR5645c  ath-miR5645d  ath-miR5645f  ath-miR5645e |
| 37 | ath-miR5635a  ath-miR5635d  ath-miR5635b  ath-miR5635c | 38 | ath-miR5630a  ath-miR5630b |
| 39 | ath-miR5630a  ath-miR5630b | 40 | ath-miR5642a  ath-miR5642b |
| 41 | ath-miR5643a  ath-miR5643b | 42 | ath-miR5638a  ath-miR5638b |
| 43 | ath-miR5649a  ath-miR5649b | 44 | ath-miR5595a  ath-miR5995b |
| 45 | ath-miR5998a  ath-miR5998b | 46 | ath-miR8167a  ath-miR8167b  ath-miR8167c  ath-miR8167d  ath-miR8167e  ath-miR8167f |
