# Supplementary material for: Inferring plant microRNA functional similarity using a weighted protein-protein interaction network
Source: BMC Bioinformatics. 2015 Nov 4;16:361. doi: 10.1186/s12859-015-0789-4 (PMC4634583; doi:10.1186/s12859-015-0789-4)
Supplement: Additional file 2: Table S2. — The clusters of the A. thaliana miRNAs. (DOCX 18 kb) [file 12859_2015_789_MOESM2_ESM.docx]

| Table S2. The clusters of *A.thaliana* miRNAs | | | |
| --- | --- | --- | --- |
| Cluster No. | miRNA | Cluster No. | miRNA |
| 1 | ath-miR157a-5p  ath-miR157b-5p | 2 | ath-miR169d  ath-miR169e |
| 3 | ath-miR395a  ath-miR395b  ath-miR395c | 4 | ath-miR395d  ath-miR395e  ath-miR395f |
| 5 | ath-miR400  ath-miR5654-5p | 6 | ath-miR774a  ath-miR774b-5p  ath-miR859 |
| 7 | ath-miR781a  ath-miR781b | 8 | ath-miR833a-5p  ath-miR833b |
| 9 | ath-miR842  ath-miR846-3p | 10 | ath-miR858a  ath-miR858b |
| 11 | ath-miR4227  ath-miR5022 | 12 | ath-miR5649a  ath-miR5649b |
| 13 | ath-miR399d  ath-miR399e  ath-miR399f | 14 | ath-miR5595a  ath-miR5995b |
| 15 | ath-miR169i  ath-miR169j  ath-miR169k  ath-miR169l  ath-miR169m  ath-miR169n | 16 | ath-miR771  ath-miR851-5p |
| 17 | ath-miR782  ath-miR2934-5p  ath-miR5023 | 18 | ath-miR397b  ath-miR841a-5p  ath-miR857  ath-miR5996 |
| 19 | ath-miR447a-3p  ath-miR447b  ath-miR447c-3p | 20 | ath-miR841b-5p  ath-miR3932a |
| 21 | ath-miR845a  ath-miR845b | 22 | ath-miR850  ath-miR863-5p  ath-miR5026 |
| 23 | ath-miR867  ath-miR1887  ath-miR4239 | 24 | ath-miR5020a  ath-miR5020b |
| 25 | ath-miR162a-3p  ath-miR834 | 26 | ath-miR166c  ath-miR166d |
| 27 | ath-miR398b-3p  ath-miR398c-3p | 28 | ath-miR854a  ath-miR854c |
| 29 | ath-miR2939  ath-miR4243 | 30 | ath-miR5998a  ath-miR5998b |
