# Supplementary material for: Inferring plant microRNA functional similarity using a weighted protein-protein interaction network
Source: BMC Bioinformatics. 2015 Nov 4;16:361. doi: 10.1186/s12859-015-0789-4 (PMC4634583; doi:10.1186/s12859-015-0789-4)
Supplement: Additional file 3: Table S3. — The experimentally verified A. thaliana miRNAs that respond to different types of stress. (DOCX 29 kb) [file 12859_2015_789_MOESM3_ESM.docx]

| Table S3. The experimentally verified *A.thaliana* miRNAs in response to different stresses | | |
| --- | --- | --- |
| miRNA | Stress | Reference |
| miR156 | high-salinity | Liu HH, Tian X, Li YJ, Wu CA, Zheng CC (2008) Microarray-based analysis of stress-regulated microRNAs in Arabidopsis thaliana. Rna-a Publication of the Rna Society 14: 836-843. |
| miR159 | high-salinity |  |
| miR394 | high-salinity |  |
| miR165 | high-salinity |  |
| miR319 | high-salinity |  |
| miR393 | high-salinity |  |
| miR167 | high-salinity |  |
| miR168 | high-salinity |  |
| miR171 | high-salinity |  |
| miR396 | high-salinity |  |
| miR167 | drought |  |
| miR168 | drought |  |
| miR171 | drought |  |
| miR396 | drought |  |
| miR165 | cold |  |
| miR319 | cold |  |
| miR393 | cold |  |
| miR168 | cold |  |
| miR171 | cold |  |
| miR396 | cold |  |
| miR169 | cold |  |
| miR172 | cold |  |
| miR397 | cold |  |
| miR408 | cold |  |
| miR172a | Oil-seed Rape Mosaic Virus (ORMV)(up-regulated) | Hajdarpasic A, Ruggenthaler P (2012) Analysis of miRNA expression under stress in Arabidopsis thaliana. Bosnian Journal of Basic Medical Sciences 12: 169-176. |
| miR161 | Oil-seed Rape Mosaic Virus (ORMV)(up-regulated) |  |
| miR167a&b | Oil-seed Rape Mosaic Virus (ORMV)(up-regulated) |  |
| miR168a&b | Oil-seed Rape Mosaic Virus (ORMV)(up-regulated) |  |
| miR171a | Oil-seed Rape Mosaic Virus (ORMV)(up-regulated) |  |
| miR159 | Oil-seed Rape Mosaic Virus (ORMV)(up-regulated) |  |
| miR399d | drought(up-regulated) |  |
| miR167a&b | drought(up-regulated) |  |
| miR168a&b | drought(up-regulated) |  |
| miR171b&c | drought(up-regulated) |  |
| miR447c | drought(down-regulated) |  |
| miR173 | drought(down-regulated) |  |
| miR395 | sulfate limitation(up-regulated) | Liang G, Yang FX, Yu DQ (2010) MicroRNA395 mediates regulation of sulfate accumulation and allocation in Arabidopsis thaliana. Plant Journal 62: 1046-1057. |
| miR399 | Pi starvation(up-regulated) | Chiou TJ, Aung K, Lin SI, Wu CC, Chiang SF, et al. (2006) Regulation of phosphate homeostasis by microRNA in Arabidopsis. Plant Cell 18: 412-421. |
| miR399 | Pi starvation(up-regulated) | Hsieh LC, Lin SI, Shih ACC, Chen JW, Lin WY, et al. (2009) Uncovering Small RNA-Mediated Responses to Phosphate Deficiency in Arabidopsis by Deep Sequencing. Plant Physiology 151: 2120-2132. |
| miR778 | Pi starvation(up-regulated) |  |
| miR827 | Pi starvation(up-regulated) |  |
| miR2111 | Pi starvation(up-regulated) |  |
| miR169 | Pi starvation(repressed) |  |
| miR395 | Pi starvation(repressed) |  |
| miR398 | Pi starvation(repressed) |  |
| miR398 | Low Copper(up-regulated) | Yamasaki H, Abdel-Ghany SE, Cohu CM, Kobayashi Y, Shikanai T, et al. (2007) Regulation of copper homeostasis by micro-RNA in Arabidopsis. Journal of Biological Chemistry 282: 16369-16378. |
| miR397 | Low Copper(up-regulated) | Abdel-Ghany SE, Pilon M (2008) MicroRNA-mediated systemic down-regulation of copper protein expression in response to low copper availability in arabidopsis. Journal of Biological Chemistry 283: 15932-15945. |
| miR408 | Low Copper(up-regulated) |  |
| miR857 | Low Copper(up-regulated) |  |
| miR169 | N starvation(down-regulated) | Zhao M, Ding H, Zhu JK, Zhang FS, Li WX (2011) Involvement of miR169 in the nitrogen-starvation responses in Arabidopsis. New Phytologist 190: 906-915. |
| miR156 | N starvation(down-regulated) | Pant BD, Musialak-Lange M, Nuc P, May P, Buhtz A, et al. (2009) Identification of Nutrient-Responsive Arabidopsis and Rapeseed MicroRNAs by Comprehensive Real-Time Polymerase Chain Reaction Profiling and Small RNA Sequencing. Plant Physiology 150: 1541-1555. |
| miR167 | N starvation(down-regulated) |  |
| miR398 | N starvation(down-regulated) |  |
| miR169 | N starvation(down-regulated) | Liang G, He H, Yu DQ (2012) Identification of Nitrogen Starvation-Responsive MicroRNAs in Arabidopsis thaliana. Plos One 7: 11. |
| miR171 | N starvation(down-regulated) |  |
| miR395 | N starvation(down-regulated) |  |
| miR397 | N starvation(down-regulated) |  |
| miR398 | N starvation(down-regulated) |  |
| miR399 | N starvation(down-regulated) |  |
| miR408 | N starvation(down-regulated) |  |
| miR827 | N starvation(down-regulated) |  |
| miR857 | N starvation(down-regulated) |  |
| miR160 | N starvation(up-regulated) |  |
| miR780 | N starvation(up-regulated) |  |
| miR826 | N starvation(up-regulated) |  |
| miR842 | N starvation(up-regulated) |  |
| miR846 | N starvation(up-regulated) |  |
| miR159 | ABA-treated(up-regulated) | Khraiwesh B, Zhu JK, Zhu JH (2012) Role of miRNAs and siRNAs in biotic and abiotic stress responses of plants. Biochimica Et Biophysica Acta-Gene Regulatory Mechanisms 1819: 137-148. |
| miR393 | ABA-treated(up-regulated) |  |
| miR397b | ABA-treated(up-regulated) |  |
| miR402 | ABA-treated(up-regulated) |  |
| miR160 | ABA-treated(up-regulated) |  |
| miR417 | ABA-treated(up-regulated) |  |
| miR169 | ABA-treated(down-regulated) |  |
| miR398 | ABA-treated(down-regulated) |  |
| miR396 | drought |  |
| miR168 | drought |  |
| miR167 | drought |  |
| miR165 | drought |  |
| miR319 | drought |  |
| miR159 | drought |  |
| miR394 | drought |  |
| miR156 | drought |  |
| miR393 | drought |  |
| miR171 | drought |  |
| miR158 | drought |  |
| miR169 | drought |  |
| miR156 | salt(upregulated) |  |
| miR158 | salt(upregulated) |  |
| miR159 | salt(upregulated) |  |
| miR165 | salt(upregulated) |  |
| miR167 | salt(upregulated) |  |
| miR168 | salt(upregulated) |  |
| miR169 | salt(upregulated) |  |
| miR171 | salt(upregulated) |  |
| miR319 | salt(upregulated) |  |
| miR393 | salt(upregulated) |  |
| miR394 | salt(upregulated) |  |
| miR396 | salt(upregulated) |  |
| miR397 | salt(upregulated) |  |
| miR398 | salt(down-regulated) |  |
| miR397 | cold(upregulated) |  |
| miR169 | cold(upregulated) |  |
| miR172 | cold(upregulated) |  |
| miR165 | cold(induced) |  |
| miR166 | cold(induced) |  |
| miR393 | cold(induced) |  |
| miR396 | cold(induced) |  |
| miR408 | cold(induced) |  |
| miR395 | hypoxia(down-regulated) |  |
| miR156g | hypoxia(up-regulated) |  |
| miR157d | hypoxia(up-regulated) |  |
| miR158a | hypoxia(up-regulated) |  |
| miR159a | hypoxia(up-regulated) |  |
| miR172a,b | hypoxia(up-regulated) |  |
| miR391 | hypoxia(up-regulated) |  |
| miR775 | hypoxia(up-regulated) |  |
| miR398 | oxygen(down-regulated) |  |
| miR156 | UV-B(up-regulated) |  |
| miR157 | UV-B(up-regulated) |  |
| miR159 | UV-B(up-regulated) |  |
| miR319 | UV-B(up-regulated) |  |
| miR160 | UV-B(up-regulated) |  |
| miR165 | UV-B(up-regulated) |  |
| miR166 | UV-B(up-regulated) |  |
| miR167 | UV-B(up-regulated) |  |
| miR169 | UV-B(up-regulated) |  |
| miR170 | UV-B(up-regulated) |  |
| miR171 | UV-B(up-regulated) |  |
| miR172 | UV-B(up-regulated) |  |
| miR393 | UV-B(up-regulated) |  |
| miR398 | UV-B(up-regulated) |  |
| miR401 | UV-B(up-regulated) |  |
| miR399 | low phosphate(up-regulated) |  |
| miR395 | sulfur(induced) |  |
| miR398 | Cu(up-regulated) |  |
| miR169 | N starvation(down-regulated) |  |
| miR169b | Fe deficiency(roots) |  |
| miR169c | Fe deficiency(roots) |  |
| miR172c | Fe deficiency(roots) |  |
| miR172d | Fe deficiency(roots) |  |
| miR173 | Fe deficiency(roots) |  |
| mie394b | Fe deficiency(roots) |  |
| miR169c | Fe deficiency(shoots) |  |
| miR172c | Fe deficiency(shoots) |  |
| miR172d | Fe deficiency(shoots) |  |
| miR173 | Fe deficiency(shoots) |  |
| mie394a | Fe deficiency(shoots) |  |
| miR394b | Fe deficiency(shoots) |  |
| miR393 | bacterial PAMP(induced) |  |
| miR160 | Pst DC3000(induced) |  |
| miR167 | Pst DC3000(induced) |  |
| miR393 | Pst DC3000(induced) |  |
| miR825 | Pst DC3000(down-regulated) |  |
| miR399 | Phosphate-Starvation Response | Fujii H, Chiou TJ, Lin SI, Aung K, Zhu JK (2005) A miRNA involved in phosphate-starvation response in Arabidopsis. Current Biology 15: 2038-2043. |
| miR156 | the flowering time regulatory network | Spanudakis E, Jackson S (2014) The role of microRNAs in the control of flowering time. Journal of Experimental Botany 65: 365-380. |
| miR172 | the flowering time regulatory network |  |
| miR159 | the flowering time regulatory network |  |
| miR319 | the flowering time regulatory network |  |
| miR390 | the flowering time regulatory network |  |
| miR399 | the flowering time regulatory network |  |
| miR159a | Fe deficiency | Kong WW, Yang ZM (2010) Identification of iron-deficiency responsive microRNA genes and cis-elements in Arabidopsis. Plant Physiology and Biochemistry 48: 153-159. |
| miR169b | Fe deficiency |  |
| miR169c | Fe deficiency |  |
| miR172c | Fe deficiency |  |
| miR172d | Fe deficiency |  |
| miR173 | Fe deficiency |  |
| miR394a | Fe deficiency |  |
| miR394b | Fe deficiency |  |
| miR159 | abscisic acid (ABA) | Reyes JL, Chua NH (2007) ABA induction of miR159 controls transcript levels of two MYB factors during Arabidopsis seed germination. Plant Journal 49: 592-606. |
| miR398 | high-salinity | Jagadeeswaran G, Saini A, Sunkar R (2009) Biotic and abiotic stress down-regulate miR398 expression in Arabidopsis. Planta 229: 1009-1014. |
| miR398 | biotic stress (P. syringae) |  |
| miR398 | ozone |  |
| miR167 | the diurnal cycle | Sire C, Moreno AB, Garcia-Chapa M, Lopez-Moya JJ, Segundo BS (2009) Diurnal oscillation in the accumulation of Arabidopsis microRNAs, miR167, miR168, miR171 and miR398. Febs Letters 583: 1039-1044. |
| miR168 | the diurnal cycle |  |
| miR171 | the diurnal cycle |  |
| miR398 | the diurnal cycle |  |
| miR827 | phosphate-starvation response(induced) | Kant S, Peng MS, Rothstein SJ (2011) Genetic Regulation by NLA and MicroRNA827 for Maintaining Nitrate-Dependent Phosphate Homeostasis in Arabidopsis. Plos Genetics 7: 11. |
| miR398 | Cu/zinc (Zn) oxidative stress tolerance (copper (Cu)) | Sunkar R, Kapoor A, Zhu JK (2006) Posttranscriptional induction of two Cu/Zn superoxide dismutase genes in Arabidopsis is mediated by downregulation of miR398 and important for oxidative stress tolerance. Plant Cell 18: 2051-2065. |
| miR399 | P limitation(induced) | Pant BD, Musialak-Lange M, Nuc P, May P, Buhtz A, et al. (2009) Identification of Nutrient-Responsive Arabidopsis and Rapeseed MicroRNAs by Comprehensive Real-Time Polymerase Chain Reaction Profiling and Small RNA Sequencing. Plant Physiology 150: 1541-1555. |
| miR778 | phosphate-starvation(induced) |  |
| miR827 | phosphate-starvation(induced) |  |
| miR398a | phosphate-starvation(down-regulated) |  |
| miR398a | N starvation(down-regulated) |  |
| miR398a | C limitation(down-regulated) |  |
| miR169 | N limitation(down-regulated) |  |
| miR169 | P limitation(down-regulated) |  |
| miR447 | P limitation(induced) |  |
| miR408 | P limitation(induced) |  |
| miR2111 | P limitation(induced) |  |
| miR397 | copper limitation(induced) | Abdel-Ghany SE, Pilon M (2008) MicroRNA-mediated systemic down-regulation of copper protein expression in response to low copper availability in arabidopsis. Journal of Biological Chemistry 283: 15932-15945. |
| miR408 | copper limitation(induced) |  |
| miR857 | copper limitation(induced) |  |
| miR394 | salt(induced) | Song JB, Gao S, Sun D, Li H, Shu XX, et al. (2013) miR394 and LCR are involved in Arabidopsis salt and drought stress responses in an abscisic acid-dependent manner. Bmc Plant Biology 13: 16. |
| miR394 | drought(induced) |  |
| miR394 | ABA(induced) |  |
| miR393 | N limitation(induced) | Vidal EA, Araus V, Lu C, Parry G, Green PJ, et al. (2010) Nitrate-responsive miR393/AFB3 regulatory module controls root system architecture in Arabidopsis thaliana. Proceedings of the National Academy of Sciences of the United States of America 107: 4477-4482. |
| miR393 | cold(up-regulated) | Sunkar R, Zhu JK (2004) Novel and stress-regulated microRNAs and other small RNAs from Arabidopsis. Plant Cell 16: 2001-2019. |
| miR393 | drought(up-regulated) |  |
| miR393 | salt(up-regulated) |  |
| miR393 | ABA(up-regulated) |  |
| miR397b | cold(up-regulated little) |  |
| miR397b | drought(up-regulated little) |  |
| miR397b | salt(up-regulated little) |  |
| miR397b | ABA(up-regulated little) |  |
| miR402 | cold(up-regulated little) |  |
| miR402 | drought(up-regulated little) |  |
| miR402 | salt(up-regulated little) |  |
| miR402 | ABA(up-regulated little) |  |
| miR319c | cold(up-regulated) |  |
| miR157 | TMV-Cg virus(induced) | Tagami Y, Inaba N, Kutsuna N, Kurihara Y, Watanabe Y (2007) Specific enrichment of miRNAs in Arabidopsis thaliana infected with Tobacco mosaic virus. DNA Research 14: 227-233. |
| miR163 | TMV-Cg virus(upregulated) |  |
| miR164 | TMV-Cg virus(upregulated) |  |
| miR167 | TMV-Cg virus(upregulated) |  |
| miR166 | TMV-Cg virus(induced) |  |
| miR169 | TMV-Cg virus(induded) |  |
| miR171 | TMV-Cg virus(induced) |  |
| miR172 | TMV-Cg virus(induced) |  |
| miR319 | TMV-Cg virus(induced) |  |
| miR391 | TMV-Cg virus(induced) |  |
| miR823 | TMV-Cg virus(induced) |  |
| miR824 | TMV-Cg virus(induced) |  |
| miR169a,b,d,e,f | ABA(down-regulated) | Li YJ, Fu YR, Ji LS, Wu CA, Zheng CC (2010) Characterization and expression analysis of the Arabidopsis mir169 family. Plant Science 178: 271-280. |
| miR169b,d,e,f,i/j,k/l | heat stress(up-regulated) |  |
| miR169a,c,e | cold(up-regulated)(slight) |  |
| miR169a,c | salt(up-regulated)(slight) |  |
| miR169a | nitrogen-starvation(down-regulated) | Zhao M, Ding H, Zhu JK, Zhang FS, Li WX (2011) Involvement of miR169 in the nitrogen-starvation responses in Arabidopsis. New Phytologist 190: 906-915. |
| miR172e | drought(up-regulated) | Han YY, Zhang X, Wang YF, Ming F (2013) The Suppression of WRKY44 by GIGANTEA-miR172 Pathway Is Involved in Drought Response of Arabidopsis thaliana. Plos One 8: 16. |
