# Supplementary material for: Inferring plant microRNA functional similarity using a weighted protein-protein interaction network
Source: BMC Bioinformatics. 2015 Nov 4;16:361. doi: 10.1186/s12859-015-0789-4 (PMC4634583; doi:10.1186/s12859-015-0789-4)
Supplement: Additional file 5: Table S4. — The complete results for the miRNAs responding to high-salt conditions and TMV-Cg stress. (DOCX 17 kb) [file 12859_2015_789_MOESM5_ESM.docx]

| Table S4. The complete results of miRNAs in response to high-salt and TMV-Cg stress | | | |
| --- | --- | --- | --- |
| high-salt results | | TMV-Cg results | |
| miRNA | Score | miRNA | Score |
| ath-miR418 | 0.931773 | ath-miR165 | 1 |
| ath-miR166 | 0.928537 | ath-miR156 | 0.938794 |
| ath-miR160 | 0.907914 | ath-miR418 | 0.931773 |
| ath-miR8177 | 0.899507 | ath-miR160 | 0.907914 |
| ath-miR841 | 0.891999 | ath-miR8177 | 0.899507 |
| ath-miR169 | 0.81608 | ath-miR841 | 0.891999 |
| ath-miR5024 | 0.806945 | ath-miR5024 | 0.806945 |
| ath-miR858 | 0.801618 | ath-miR393 | 0.791004 |
| ath-miR162 | 0.7901 | ath-miR162 | 0.7901 |
| ath-miR827 | 0.781275 | ath-miR8171 | 0.776043 |
| ath-miR4239 | 0.753506 | ath-miR5015 | 0.763033 |
| ath-miR5015 | 0.749908 | ath-miR4239 | 0.748898 |
| ath-miR5634 | 0.740807 | ath-miR5652 | 0.73535 |
| ath-miR847 | 0.737371 | ath-miR8183 | 0.731316 |
| ath-miR829 | 0.735096 | ath-miR825 | 0.723367 |
| ath-miR426 | 0.732452 | ath-miR8166 | 0.718322 |
| ath-miR824 | 0.72438 | ath-miR831 | 0.715816 |
| ath-miR5017 | 0.719864 | ath-miR426 | 0.707734 |
| ath-miR851 | 0.716707 | ath-miR780 | 0.707135 |
| ath-miR863 | 0.712811 | ath-miR863 | 0.706336 |
| ath-miR831 | 0.710787 | ath-miR5020 | 0.705098 |
| ath-miR5998 | 0.710698 | ath-miR407 | 0.696193 |
| ath-miR5662 | 0.708089 | ath-miR168 | 0.693561 |
| ath-miR833 | 0.704464 | ath-miR2111 | 0.693239 |
| ath-miR5648 | 0.700799 | ath-miR855 | 0.693004 |
| ath-miR828 | 0.700447 | ath-miR8165 | 0.690203 |
| ath-miR157 | 0.700423 | ath-miR833 | 0.689801 |
| ath-miR8171 | 0.698301 | ath-miR5017 | 0.683035 |
| ath-miR3933 | 0.695274 | ath-miR847 | 0.682425 |
| ath-miR2111 | 0.693239 | ath-miR5648 | 0.682226 |
| ath-miR4227 | 0.692643 | ath-miR5650 | 0.68135 |
| ath-miR780 | 0.688849 | ath-miR8173 | 0.678144 |
| ath-miR866 | 0.686231 | ath-miR159 | 0.678036 |
| ath-miR2934 | 0.685893 | ath-miR1888 | 0.674695 |
| ath-miR172 | 0.682234 | ath-miR774 | 0.671075 |
| ath-miR5638 | 0.678826 | ath-miR5647 | 0.671052 |
| ath-miR5643 | 0.677538 | ath-miR865 | 0.669125 |
| ath-miR862 | 0.676905 | ath-miR396 | 0.668824 |
| ath-miR5647 | 0.676514 | ath-miR829 | 0.668772 |
| ath-miR5664 | 0.676425 | ath-miR5998 | 0.66769 |
| ath-miR1888 | 0.674695 | ath-miR399 | 0.666716 |
| ath-miR774 | 0.674606 | ath-miR837 | 0.666165 |
| ath-miR825 | 0.673463 | ath-miR5634 | 0.661649 |
| ath-miR5650 | 0.670655 | ath-miR4221 | 0.659473 |
| ath-miR865 | 0.66745 | ath-miR779 | 0.65639 |
| ath-miR5020 | 0.665453 | ath-miR2937 | 0.650488 |
| ath-miR8183 | 0.662215 | ath-miR870 | 0.648865 |
| ath-miR864 | 0.656294 | ath-miR858 | 0.648717 |
| ath-miR8165 | 0.655586 | ath-miR8182 | 0.648056 |
| ath-miR843 | 0.653968 | ath-miR5662 | 0.647865 |
| ath-miR4221 | 0.652923 | ath-miR776 | 0.647585 |
| ath-miR773 | 0.651913 | ath-miR860 | 0.647402 |
| ath-miR5025 | 0.648658 | ath-miR5638 | 0.647312 |
| ath-miR869 | 0.64834 | ath-miR851 | 0.642088 |
| ath-miR5026 | 0.6482 | ath-miR4243 | 0.641952 |
| ath-miR8182 | 0.648056 | ath-miR866 | 0.640281 |
| ath-miR860 | 0.647402 | ath-miR775 | 0.639857 |
| ath-miR776 | 0.645726 | ath-miR3434 | 0.638184 |
| ath-miR5637 | 0.645565 | ath-miR413 | 0.636581 |
| ath-miR5999 | 0.645179 | ath-miR862 | 0.636047 |
| ath-miR837 | 0.642247 | ath-miR827 | 0.635664 |
| ath-miR8166 | 0.640363 | ath-miR846 | 0.633415 |
| ath-miR407 | 0.640228 | ath-miR5641 | 0.632055 |
| ath-miR5641 | 0.639048 | ath-miR826 | 0.628389 |
| ath-miR4228 | 0.636743 | ath-miR773 | 0.625306 |
| ath-miR413 | 0.636581 | ath-miR414 | 0.623831 |
| ath-miR870 | 0.635627 | ath-miR447 | 0.622145 |
| ath-miR5014 | 0.634452 | ath-miR854 | 0.622074 |
| ath-miR846 | 0.633415 | ath-miR859 | 0.622033 |
| ath-miR3434 | 0.632883 | ath-miR5631 | 0.620367 |
| ath-miR2937 | 0.632001 | ath-miR5025 | 0.620325 |
| ath-miR779 | 0.630373 | ath-miR390 | 0.620265 |
| ath-miR5631 | 0.629955 | ath-miR836 | 0.617945 |
| ath-miR834 | 0.626112 | ath-miR5643 | 0.61732 |
| ath-miR836 | 0.623879 | ath-miR2934 | 0.616501 |
| ath-miR164 | 0.621207 | ath-miR869 | 0.615734 |
| ath-miR4243 | 0.61934 | ath-miR781 | 0.61215 |
| ath-miR826 | 0.618418 | ath-miR5633 | 0.612086 |
| ath-miR855 | 0.616353 | ath-miR782 | 0.60925 |
| ath-miR830 | 0.615646 | ath-miR398 | 0.607585 |
| ath-miR390 | 0.614598 | ath-miR3933 | 0.607458 |
| ath-miR158 | 0.614251 | ath-miR832 | 0.606103 |
| ath-miR1886 | 0.614214 | ath-miR5637 | 0.605351 |
| ath-miR867 | 0.613959 | ath-miR830 | 0.605291 |
| ath-miR8178 | 0.612551 | ath-miR828 | 0.597279 |
| ath-miR5633 | 0.611059 | ath-miR8170 | 0.595222 |
| ath-miR447 | 0.609362 | ath-miR834 | 0.594775 |
| ath-miR852 | 0.604494 | ath-miR1886 | 0.593699 |
| ath-miR414 | 0.604325 | ath-miR5664 | 0.593204 |
| ath-miR5660 | 0.599883 | ath-miR5999 | 0.592788 |
| ath-miR398 | 0.599326 | ath-miR8178 | 0.592416 |
| ath-miR399 | 0.598189 | ath-miR5653 | 0.591899 |
| ath-miR8173 | 0.597248 | ath-miR4228 | 0.589426 |
| ath-miR161 | 0.596236 | ath-miR5014 | 0.587512 |
| ath-miR8170 | 0.595222 | ath-miR5663 | 0.587311 |
| ath-miR8179 | 0.594211 | ath-miR2936 | 0.58646 |
| ath-miR5653 | 0.591899 | ath-miR4227 | 0.584184 |
| ath-miR5645 | 0.588921 | ath-miR8179 | 0.582772 |
| ath-miR781 | 0.588309 | ath-miR864 | 0.581646 |
| ath-miR854 | 0.588299 | ath-miR472 | 0.581454 |
| ath-miR844 | 0.584472 | ath-miR2938 | 0.580433 |
| ath-miR2938 | 0.583979 | ath-miR394 | 0.578429 |
| ath-miR391 | 0.583143 | ath-miR5016 | 0.574401 |
| ath-miR5028 | 0.582966 | ath-miR778 | 0.573545 |
| ath-miR5657 | 0.582777 | ath-miR5019 | 0.573016 |
| ath-miR782 | 0.581181 | ath-miR5595 | 0.571132 |
| ath-miR5016 | 0.57894 | ath-miR5995 | 0.571132 |
| ath-miR5654 | 0.578096 | ath-miR843 | 0.570938 |
| ath-miR2936 | 0.577858 | ath-miR5658 | 0.568568 |
| ath-miR5652 | 0.576954 | ath-miR397 | 0.564129 |
| ath-miR5630 | 0.575522 | ath-miR5021 | 0.56363 |
| ath-miR415 | 0.574588 | ath-miR835 | 0.560595 |
| ath-miR2112 | 0.574431 | ath-miR5657 | 0.558023 |
| ath-miR3932 | 0.573766 | ath-miR5645 | 0.55731 |
| ath-miR5019 | 0.573016 | ath-miR415 | 0.557193 |
| ath-miR472 | 0.572548 | ath-miR867 | 0.556416 |
| ath-miR832 | 0.572091 | ath-miR838 | 0.552162 |
| ath-miR5595 | 0.571132 | ath-miR2933 | 0.550703 |
| ath-miR5995 | 0.571132 | ath-miR856 | 0.550216 |
| ath-miR5635 | 0.568266 | ath-miR5028 | 0.549077 |
| ath-miR859 | 0.56641 | ath-miR5630 | 0.548648 |
| ath-miR397 | 0.564129 | ath-miR161 | 0.548622 |
| ath-miR856 | 0.55713 | ath-miR158 | 0.546263 |
| ath-miR835 | 0.556956 | ath-miR5660 | 0.54588 |
| ath-miR5658 | 0.555943 | ath-miR8181 | 0.544934 |
| ath-miR838 | 0.54764 | ath-miR5029 | 0.540461 |
| ath-miR5632 | 0.546919 | ath-miR2112 | 0.537135 |
| ath-miR8172 | 0.543943 | ath-miR5013 | 0.535373 |
| ath-miR775 | 0.540524 | ath-miR403 | 0.535314 |
| ath-miR5013 | 0.538558 | ath-miR5666 | 0.532679 |
| ath-miR5021 | 0.538367 | ath-miR3932 | 0.531364 |
| ath-miR8181 | 0.537931 | ath-miR8172 | 0.51376 |
| ath-miR403 | 0.535314 | ath-miR5635 | 0.509554 |
| ath-miR868 | 0.534681 | ath-miR5640 | 0.505268 |
| ath-miR5665 | 0.533086 | ath-miR771 | 0.502007 |
| ath-miR778 | 0.529324 | ath-miR419 | 0.501295 |
| ath-miR5666 | 0.527711 |  |  |
| ath-miR4245 | 0.521535 |  |  |
| ath-miR822 | 0.519937 |  |  |
| ath-miR5640 | 0.519619 |  |  |
| ath-miR3440 | 0.516529 |  |  |
| ath-miR5663 | 0.515902 |  |  |
| ath-miR419 | 0.510114 |  |  |
| ath-miR771 | 0.502007 |  |  |
| ath-miR5642 | 0.501804 |  |  |
